# Network meta-analysis of invasive treatment for early-stage osteonecrosis of the femoral head

**DOI:** 10.1186/s13018-023-04513-x

**Published:** 2024-01-03

**Authors:** Yingchun Li, Xiuying Ma, Bo Dong, Yue Li, Zhuang Liang

**Affiliations:** Pain Ward of Rehabilitation Department, Honghui Hospital, Xi’an Jiaotong University, No. 555 Youyi East Road, Beilin District, Xi’an, 710054 Shaanxi Province People’s Republic of China

**Keywords:** Ischemic necrosis, Osteonecrosis, Core decompression, Surgical treatment, Meta-analysis

## Abstract

**Background:**

Osteonecrosis of the femoral head (ONFH) is a common disabling disease in orthopedics. Blocking the progression of ONFH in the early stage is essential for avoiding total hip replacement.

**Purposes:**

The purpose of this study is to evaluate the effect of invasive treatment on early-stage ONFH.

**Methods:**

According to the PRISMA guidelines, relevant English databases were searched in August 2022 to collect published research. Extract result indicators and conduct network meta-analysis using R software.

**Results:**

A total of 15 RCTs were included. All patients were diagnosed with early-stage ONFH. The surface under the cumulative ranking curve (SUCRA) showed that CD + BMMSC and CD + PRP were the most effective in improving HHS. The results of the league table showed that CD + BMMSC was superior to CD alone. Meanwhile, the SUCRA for FR showed that CD + BG + BMMSC was the most likely to be the most effective in reducing FR. The league table revealed that CD + BG, CD + BG + BMMSC, and CD + BMMSC were superior to CD alone, with statistically significant differences.

**Conclusion:**

Considering the HHS and FR, CD + BMMSC may be the optimal treatment option to effectively delay the progression of ONFH and restore the postoperative function of patients.

*Registration Number*: The study protocol has been registered on the PROSPERO platform (CRD42023380169).

**Supplementary Information:**

The online version contains supplementary material available at 10.1186/s13018-023-04513-x.

## Introduction

Osteonecrosis of the femoral head (ONFH) is a progressive hip joint disease. Early-stage ONFH refers to the ischemic necrosis of bone tissue of the femoral head, which will then progress to femoral head collapse and hip joint dysfunction [42]. It usually occurs in adults aged 20–40, and is characterized by the death, decomposition, and collapse of cells and bone tissue of the femoral head. Early ONFH is usually considered as a progressive disease, which may be asymptomatic or cause slight discomfort. According to statistical investigations, there are about 8.12 million patients with ONFH in China, and the age of onset is mostly 20–55 years. Its prevalence rate is higher in males than in females [46], and its incidence rate is increasing yearly. In the USA, it affects more than 20,000 to 30,000 additional patients every year [14]. In Germany, its annual incidence rate is 0.01%, affecting about 5000 to 7000 people [2]. If it is not treated in time, the necrotic bone tissue will be irreversibly damaged, and about 70–80% of patients will develop secondary hip arthritis, which will eventually require total hip replacement (THR) [20]. THR is an effective method to treat ONFH. However, due to the high cost and heavy economic burden, this artificial replacement is difficult to last for a lifetime for young patients, so early treatment is critical [24, 42]. In the early stage, proper treatment can effectively delay the progression of the disease, avoid large-scale necrosis of bone tissue, and ultimately reduce the patients’ need for hip replacement surgery [2]. Various approaches can be used to promote the repair and regeneration of necrotic bones, and concurrently alleviate the pain of patients and improve the function of hip joint. Particularly, invasive treatment for this disease at the early stage has attracted much attention in clinical settings [4].

Hua et al.’s research showed that core decompression (CD) can be used as an effective means to treat early-stage ONFH [17, 30]. More treatment methods have been developed on this basis, including autologous bone grafting (BG), platelet-rich plasma (PRP) perfusion, superselective arterial infusion (SAI) and stem cell therapy, and their combined application [32]. These methods can treat early-stage ONFH by improving blood supply, repairing bone tissue, and relieving pain. In the specific application, the safety and efficacy of these methods are still controversial, and there is a lack of systematic evaluation and evidence support [27]. Therefore, an in-depth understanding and comparison of different invasive treatment methods is helpful for providing more scientific and effective treatment options for patients with ONFH.

Network meta-analysis (NMA) is an analysis method based on traditional meta-analysis, which provides both direct comparison and indirect comparison and integrates their results into a network structure. Compared with traditional meta-analysis, NMA provides more comprehensive data and more statistical analysis options, which enables us to compare and rank the effects of different treatment interventions. It can make a direct comparison and estimate the relative effects of different interventions. Therefore, NMA plays an important role in integrating and synthesizing different research results and is widely used in current medical research.

The purpose of this study is to systematically evaluate and compare the effects of the above invasive methods in the treatment of early-stage ONFH, so as to provide more reliable clinical evidence and treatment guidance for clinicians to make a more reliable decision to improve the treatment effect and quality of life of patients to the greatest extent.

## Data and methods

The current study was conducted in accordance with the Preferred Reporting Items for Systematic Reviews and Meta-Analyses (PRISMA) statement [18]. The study protocol has been registered on the PROSPERO platform (CRD42023380169) [45].

### Literature retrieval

Published works of literature about the treatment of early-stage ONFH were retrieved in English databases including PubMed, EMBASE, Cochrane Library, and Web of Science from the database inception to August 2022. The following English keywords were used: Aseptic Necrosis of Femur Head, Avascular Necrosis of Femur Head, femoral head necrosis, femoral head osteonecrosis, Femur Head Necroses, Femur Head Necrosis, femur head osteonecrosis, Ischemic Necrosis of Femoral Head, juvenile femur head necrosis, ONFH, osteonecrosis of the femoral head, Osteonecrosis of the femur head. The MeSH thesaurus was queried, and both the subject words and free words were used for the search. Meanwhile, the references of related literature were searched manually.

### Literature screening

Literature screening was conducted by two researchers independently, and any discrepancies would be resolved by a third researcher. The inclusion criteria of screening were as follows: (1) Study type: prospective case–control study or randomized controlled trial (RCT); (2) Participants: subjects with early non-traumatic ONFH (patients who did not receive a total hip replacement); (3) Interventions: surgical approaches for ONFH; and (4) Outcome measures: Harris hip score (HHS) and total hip replacement rate. Exclusion criteria were as follows: 1) Participants: subjects without early-stage ONFH; 2) without comparison between the above treatments, or with insufficient information to extract relevant data; and 3) written in non-English; and (4) duplicate publications, reviews, systematic evaluations, meta-analyses, case reports, animal experiments, etc.

### Data extraction

Two researchers independently read the abstracts and full texts to determine the eligibility of relevant literature. The following information was extracted from the included literature: the first author, publication year, country and region, patient's age and gender, sample size, ONFH stage, outcomes, HHS and final failure rate (FR) (The final surgical failure rate is defined as the need for hip replacement surgery or progression to a high-risk stage after surgery, such as femoral head collapse), etc. The differences were resolved through consultation with a third researcher. HHS with the most adjustment factors were extracted in the case of multiple HHSs with different adjustment factors. If the HHS could not be directly obtained, it would be indirectly calculated according to Tierney et al.’s [39] method or extracted from the survival curve using Engauge Digitizer software (version 4.1).

### Literature quality evaluation

According to the Cochrane risk-of-bias tool, two independent researchers evaluated the methodological quality of the included documents, all of which were in English. Evaluation criteria were: (1) Whether the random allocation method is correct; (2) Whether the allocation concealment scheme is correct; (3) Whether the doctors and subjects were blinded to the intervention; (4) Whether the evaluator was blinded to the result evaluation; (5) Whether the data of expected outcome indicators and follow-up reports are complete; (6) Whether there is a selective report; (7) Other biases, for example, whether the data included in the literature are suspected of fraud, whether the baseline of the literature is consistent, and whether there is any conflict of interest involved in the research.

### Statistical processing

Utilizing the “gemtc” package in R software (version 4.1.2), statistical analysis was performed based on the Markov Chain Monte Carlo (MCMC) method. The results were described by combined risk ratio (RR)/ mean difference (MD) with corresponding 95% confidence intervals (CIs), and the significance level was analyzed according to whether 95% CI contains an invalid value (contains the value 1). The node splitting method was used to evaluate the local inconsistency, and a *p* value less than 0.05 indicates statistically significant inconsistency. Heterogeneity was evaluated using the heterogeneity test. *I*^2^ < 50% indicates negligible heterogeneity, *I*^2^ value of 50–70% may represent acceptable heterogeneity, while *I*^2^ > 70% indicates that there is substantial heterogeneity. Finally, the league table of pairwise comparison of the treatment methods after combined analysis was obtained. The ranking probability of each treatment was estimated and the SUCRA value was calculated.

## Results

### Literature retrieval

**Fig. 1:**
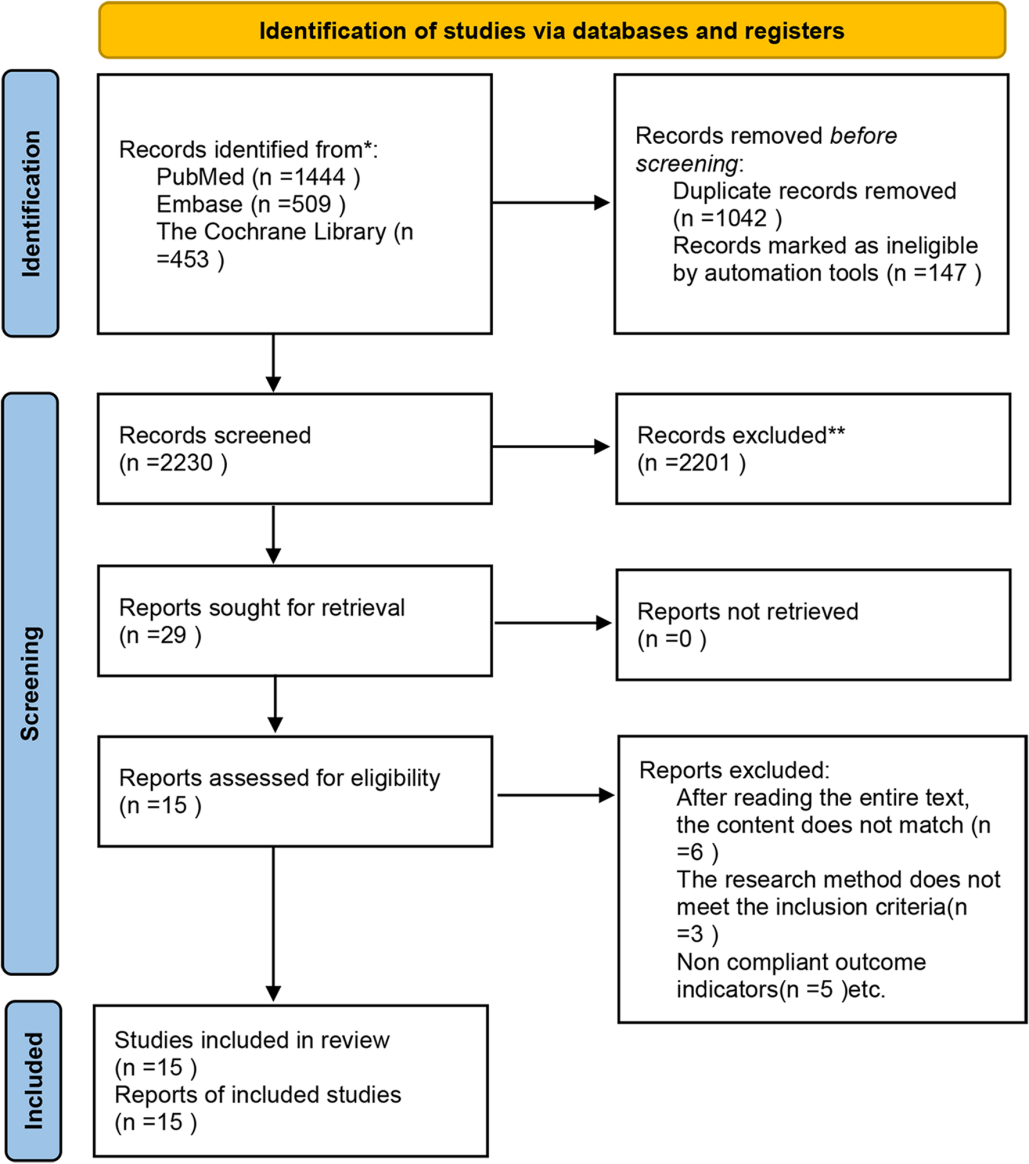
PRISMA 2020 flow diagram for new systematic reviews

A total of 3448 articles were retrieved, including 1042 duplicates and 147 letters or comments. 2230 articles were excluded by screening the titles and abstracts, and the remaining 29 articles were searched for full texts and evaluated for eligibility. Finally, 15 RCTs were included. The specific process is shown in Fig. 1. The treatment strategies encompassed core decompression, bone grafting, platelet-rich plasma perfusion, superselective arterial infusion, stem cells, and their combined application. The endpoints of this study include the HHS and FR. The study selection process is presented in Fig. 1.

### Basic characteristics of included studies

A total of 15 articles were included [1, 6–8, 12, 13, 16, 23, 25, 34, 37, 38, 43, 47, 48], involving 605 patients with 743 hips. All patients were diagnosed with early-stage ONFH. Among them, 298 hips of the patients were treated with CD alone; 222 hips were treated with CD combined with bone marrow mesenchymal stem cells (BMMSC); 99 hips were treated with CD combined with bone grafting (BG); 45 hips were treated with bone grafting alone; 29 hips were treated with CD combined with superselective arterial infusion (SAI); 53 hips were treated with BMMSC alone; 25 hips were treated with CD combined with platelet-rich plasma (PRP), and 25 hips were treated with CD combined with BMMSC. The basic characteristics of the included studies are detailed in Table 1. Figure 3a, b shows evidence network diagrams for different outcomes.

**Table 1:** The basic characteristics of the included studies

Author	Year	Region	Population	Age (mean, SD)	Gender (♀, ♂)	Ficat grade	ARCO stage	HHS (mean, SD)	Comparisons
Cao	2017	China	21	31, 6	5, 16	NA	I, 5/II, 26/III, 11	60, 8.03	CD versus BG
Aggarwal	2020	India	40	NA	3, 37	I, 13/II, 40	I, 13/II, 40	63.49, 8.04	CD versus CD + PRP
Li	2016	China	39	36.5	12, 27	I, 21/II, 26	NA	63.71, 3.58	BG versus CD + BG
Gangji	2011	Belgium	19	45.7, 2.8	10, 9	NA	I, 4/II, 20	NA	CD versus CD + BMMSC
Fu	2022	China	40	38.98, 3.95	11, 29	NA	I, 27/II, 13	77.73, 2.67	CD versus CD + BMMSC
Ma	2014	China	43	35.22, 9.66	11, 28	I, 7/II, 32/III, 10	NA	NA	CD + BG versus CD + BG + BMMSC
Hauzeur	2018	Belgium	38	48.85, 3.09	11, 27	NA	III, 46	NA	CD versus CD + BMMSC
Rastogi	2013	India	40	33.84, 7.36	11, 29	NA	I, 14/II, 22/III, 24	46.92, 1.70	CD + BG versus CD + BMMSC
Zhao	2012	China	100	33.25, 9.18	47, 53	NA	I, 5/II, 99	42.75, 4.50	CD versus CD + BMMSC
Tabatabaee	2015	Iran	28	28.9, 9.13	9, 19	NA	I, 5/II, 16/III, 7	NA	CD versus CD + BMMSC
Sen	2012	India	40	NA	13, 27	NA	I, II, 51	65.96, 14.02	CD versus CD + BMMSC
Chang	2010	China	8	35.7	1, 7	NA	II, 14/III, 2	56.19, 8.60	CD versus CD + BMMSC
Chen	2016	China	71	38.39, 10.11	27, 44	I, II, 51/III, 20	NA	39.63, 5.9	CD versus CD + SAI
Wang	2017	China	30	NA	11, 19	NA	I, 17/II, 22	NA	CD versus CD + BG
Yu	2014	China	48	37.77, 8.39	33, 15	NA	I, 9/II, 41/III, 23	52.96, 6.88	CD versus CD + BMMSC

*CD* Core decompression, *BG* Bone grafting, *PRP* Platelet-rich plasma, *BMMSC* Bone marrow mesenchymal stem cells, *SAI* Superselective arterial infusion

### Results of the risk of bias assessment for the included studies

**Fig. 2:**
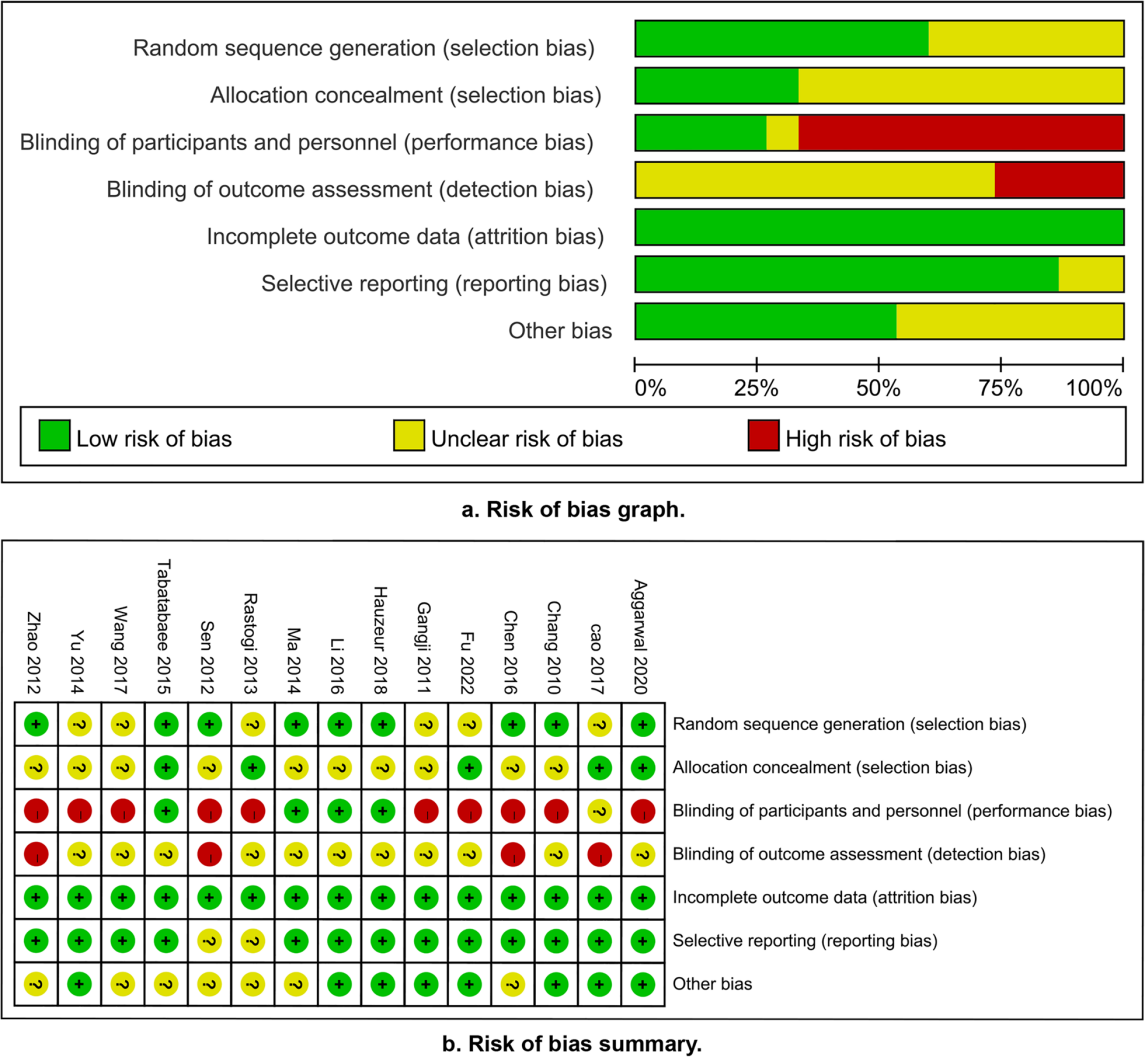
Results of the risk of bias assessment for the included studies

A risk assessment of the included studies was conducted, and the results showed that the overall quality was moderate. The main reason for the impaired quality of evidence was that all interventions were surgery, making it difficult to find a suitable blinding method during treatment. Figure 2 shows the results of the bias risk assessment included in the study.

### Bayesian network meta-analysis of HHS

**Fig. 3:**
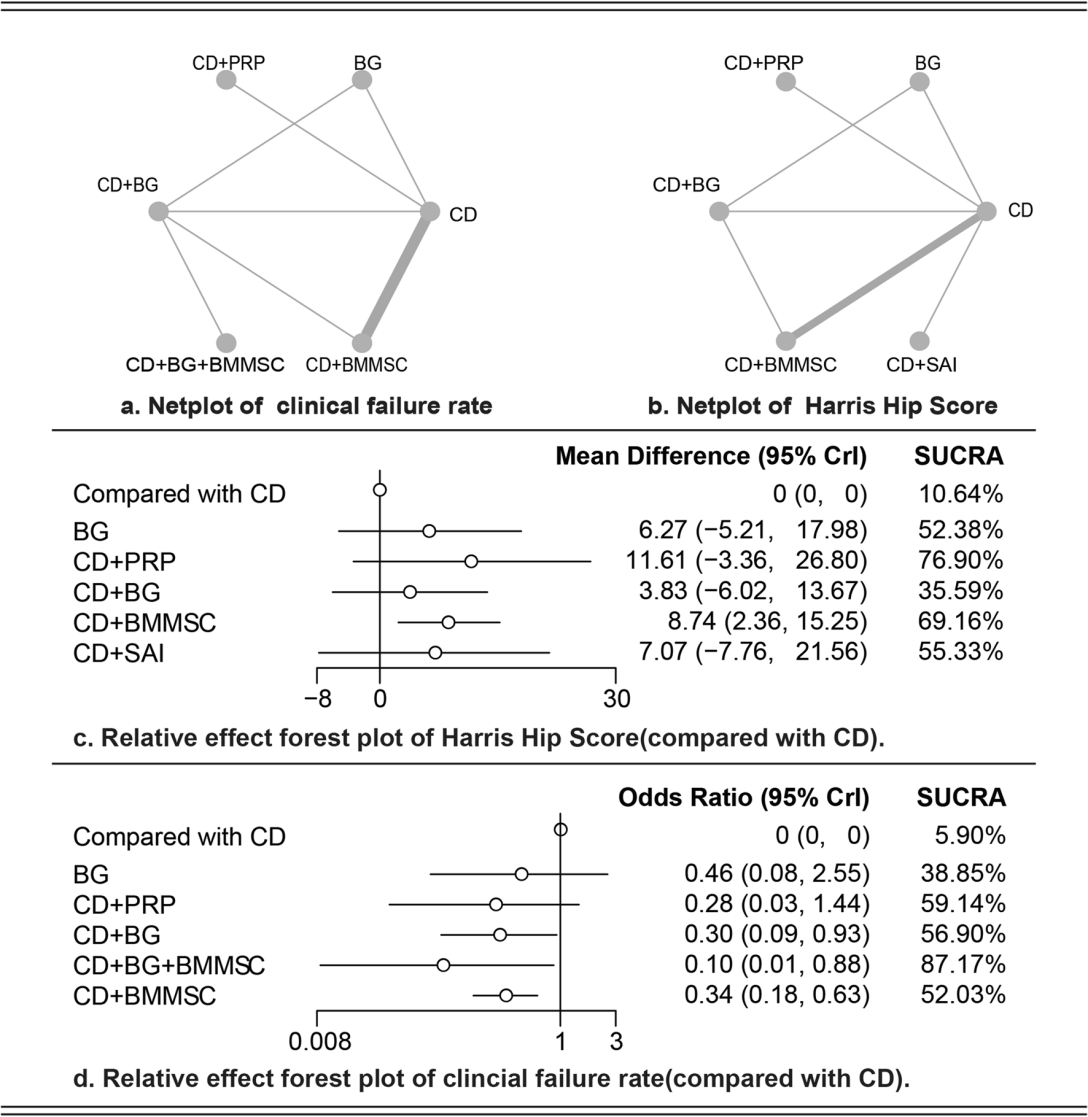
Summary of the result of network meta-analysis

Eleven studies reported HHS after different interventions, involving seven treatment methods. The forest plot is shown in Fig. 3c. The surface under the cumulative ranking curve (SUCRA) showed that CD + BMMSC (76.90%) and CD + PRP (69.16%) were the most effective, followed by CD + SAI (55.33%) and BG (52.38%). Finally, the league table for HHS is shown in Table 2. Although the ranking of these interventions was obtained, the league table showed that CD + BMMSC outperformed CD alone in terms of improving HHS (MD = 8.74, 95% CI 2.36, 15.25), with a statistical difference. No significant differences were observed in other comparisons.

**Table 2:** League table of the results of network meta-analysis

		**CD**	6.27 (− 5.21, 17.98)	11.61 (− 3.36, 26.80)	3.83 (− 6.02, 13.67)	**8.74 (2.36, 15.25**)	7.07 (− 7.76, 21.56)	HHS Score
	**CD**		**BG**	5.38 (− 13.63, 24.29)	− 2.44 (− 14.05, 9.03)	2.48 (− 10.07, 15.05)	0.77 (− 17.97, 19.23)	
	2.16 (0.39, 13.13)	**BG**		**CD + PRP**	− 7.83 (− 25.87, 10.18)	− 2.88 (− 19.16, 13.41)	− 4.57 (− 25.77, 16.39)	
	3.59 (0.69, 29.66)	1.69 (0.14, 24.83)	**CD + PRP**		**CD + BG**	4.91 (− 5.27, 15.21)	3.27 (− 14.57, 20.83)	
	**3.3 (1.08, 10.65)**	1.51 (0.29, 8.07)	0.91 (0.09, 7.05)	**CD + BG**		**CD + BMMSC**	− 1.66 (− 17.90, 14.14)	
	**10.13 (1.14, 116.98)**	4.68 (0.38, 71.25)	2.77 (0.14, 54.07)	3 (0.48, 26.84)	**CD + BG + BMMSC**		**CD + SAI**	
Clinical Failure Rate	**2.92 (1.58, 5.61)**	1.36 (0.21, 8.14)	0.81 (0.09, 4.84)	0.88 (0.26, 2.92)	0.29 (0.02, 2.68)	**CD + BMMSC**		

The left lower part is the league table of clinical failure rate, risk ratio and 95% credit interval are presented in it; and the right upper part is the league table of the HHS score, mean difference and 95%CrI are presented in it

*CD* Core decompression, *BG* Bone grafting, *PRP* Platelet-rich plasma, *BMMSC* Bone marrow mesenchymal stem cells, *SAI* Superselective arterial infusion

In comparison to CD alone, CD+BG, CD+BG+BMMSC, and CD+BMMSC all exhibit significant statistical significance and demonstrate superior efficacy compared to CD alone

### Bayesian network meta-analysis of FR

A total of 13 studies reported the FR of different interventions, involving 7 treatment methods. The construction model and iteration method are the same as mentioned above. The forest plot for FR is shown in Fig. 3d. The league table for FR is shown in Table 2. CD + BG [RR = 3.3, 95% CI 1.08, 10.65], CD + BG + BMMSC (RR = 10.13, 95% CI 1.14, 116.98), and CD + BMMSC (RR = 2.92, 95% CI 1.58, 5.61) outperformed CD alone in terms of FR, with statistically significant differences. The SUCRA showed that CD + BG + BMMSC (87.17%) had the highest probability to be the best intervention, while CD + PRP (59.14%), CD + BG (56.90%), and CD + BMMSC (52.03%) had similar effects.

### Inconsistency test, heterogeneity analysis, and PSIF values

We examined the PSIF values of the network meta-analysis of all outcomes, with all values of PSIF = 1, indicating a satisfactory degree of convergence. The node-splitting method was leveraged to test the local inconsistency before network meta-analysis of all outcome indicators, and the consistency was considered good (P > 0.05). At the same time, the heterogeneity analysis was carried out, with all values of *I*^2^ < 50%, indicating small and ignorable heterogeneity. The detailed results are provided in Additional file 1.

## Discussion

This meta-analysis included 15 RCTs with a total of 743 hips, involving 9 surgical schemes for early treatment of ONFH. The results showed that CD + BG + BMMSC has the lowest FR and may be effective to delay the progression of ONFH, while CD and BG alone rank last, possibly because CD or BG alone cannot provide effective physical support or effectively promote tissue regeneration of the femoral head [3]. Regarding HHS after operation, CD + BMMSC and CD + PRP rank first, suggesting that these two surgical schemes are more conducive to the functional recovery of patients after operation and may be the best treatment schemes. Considering the two outcome indicators, CD + BMMSC may be the best treatment option to effectively delay the progression of ONFH and restore the postoperative function of patients.

CD is considered to be the most commonly used surgical method for the treatment of early-stage ONFH, which can repair function and delay the progression of ONFH by effectively reducing intra-osseous pressure, accelerating bone regeneration, reversing ONFH, stimulating the formation of blood vessels around the core decompression channel, and improving blood flow [31]. CD has been demonstrated to significantly reduce the pain of hip joint in patients with early avascular necrosis of femoral head and increase the range of motion of hip joint [11]. However, another study has shown that CD is only effective in the short term but less effective in the long-term treatment and cannot avoid total hip replacement [29, 35]. Moreover, large-diameter decompression may lead to articular cartilage injury or fracture, which will increase the risk of total hip replacement [5]. Although the effect of CD alone is inconsistent, its combination with other treatment methods has shown promising results [26]. CD + BG has been reported to improve the success rate of surgery for early-stage ONFH by providing scaffolds and stem cells for bone regeneration [17, 23], but some researchers hold a negative attitude toward this method. In addition, by repositioning necrotic tissue to a non-load-bearing location, osteotomy could improve the perfusion of the necrotic area and thus delay the progression of ONFH [41]. However, due to its high THR conversion rate, higher-quality randomized controlled trials are needed to verify its efficacy [33].

With the rapid development of cell biotechnology and tissue engineering, stem cell therapy has shown promising results in the treatment of ONFH [44]. Stem cells have been proven to have the ability of self-renewal and differentiation into many cell types. Mesenchymal stem cells are the main progenitor stromal cells of bone regeneration. They can differentiate into osteocytes, adipocytes, and chondrocytes, affect bone repair and angiogenesis, and produce growth factors, which can promote blood supply to necrotic regions through paracrine effects [21, 22]. Although stem cells have great potential in the treatment of ONFH, there are still some important challenges. The source, quantity, quality, and activity of stem cells may be affected by the age, diseases, and drugs of the donors, resulting in unstable therapeutic effects, and the transplantation efficiency and survival rate of stem cells may be limited by the ischemic and necrotic environment [10]. When the combination of CD and stem cells is used to treat early-stage ONFH, stem cells can be injected through the tunnel opened by core decompression to increase the regeneration ability of bone tissue, and the environment within the bone created by core decompression can promote cell differentiation [19, 28]. Although the efficacy of stem cell grafting has been confirmed, it is not widely used in some economically underdeveloped areas due to the high cost and complex technology.

PRP is a platelet concentrate rich in various growth factors, which can promote bone healing and soft tissue repair. Samy [36] treated 40 cases of hip with CD + BG + PRP, with an average follow-up of 41 months. At the last follow-up, the necrotic area of 35 hips was reduced, the bones were healed, and cystic degeneration disappeared, indicating a good therapeutic effect. Han et al. [15] reviewed 17 studies on the treatment of ONFH with PRP. The results showed that PRP could not reverse the pathophysiological procession of ONFH, but it could induce osteoblast activity and stimulate stem cell differentiation when combined with other surgical methods. However, there is still a lack of strong evidence of its effectiveness in treating ONFH. Selective arterial infusion is mainly aimed at addressing the compromised blood supply of ONFH patients by injecting a thrombolytic agent and vasodilator directly into the femoral artery through the catheter to dredge the nutrient vessels of the femoral head, increase collateral circulation, improve the blood supply of femoral head, and repair the necrotic area. Tong et al. [40] treated 78 patients with non-traumatic ONFH with superselective arterial infusion (SAI) of bone marrow stromal cells. HHS of hip joint was significantly improved at the last follow-up compared with that before operation. DSA showed thickened medial circumflex femoral artery and increased branches, which suggested that it could effectively treat ARCO I and II non-traumatic ONFH. Selective arterial infusion is believed to be a simple, convenient, economical, and feasible method, which is suitable for primary hospitals and has fewer requirements for technology [9].

Altogether, based on the results of this meta-analysis, CD + BG + BMMSC is the most effective treatment option, whereas the high cost and complex technology hinder the widespread use of this method. Future research can focus on the laboratory qualification and technical requirements of stem cell collection, sorting, and culture system. CD + SAI is worth popularizing due to its efficacy, operability, and lower costs, but high-quality experimental research is required to provide strong evidence. CD + PRP is characterized by simple operation, safety, and effectiveness. Despite the superiority of CD + BG + BMMSC in reducing FR, further research is required to substantiate this finding due to little available data-merely one trial, in which HHS is not reported. Nonetheless, our study also furnishes novel insights into the treatment of early ONFH with the combination of multiple interventions. Future research can further explore the efficacy of combined interventions in the treatment of this disease to maximize the treatment benefits.

This systematic review includes 15 studies, all of which are randomized controlled trials. This can provide strong evidence for the effectiveness and safety of invasive treatment for early-stage ONFH. However, this meta-analysis still has some limitations: (1) The overall quality of the included literature is not high, the sample size is small, and the follow-up time of some studies was short, which may lead to potential bias. (2) The report on the outcome indicators of the included literature is incomplete, which makes it impossible to evaluate the heterogeneity. (3) The publication bias can't be evaluated because there are few articles included in this study. Based on these limitations, future research can focus on the following directions: increasing the quality and sample size of research literature, improving the report of outcome indicators, considering publication bias, and conducting multi-center and large sample research. These improvements will help to improve the reliability and popularization of the research.

## Conclusion

To sum up, CD + BMMSC may be the optimal choice to treat patients with early-stage ONFH. CD + BG, CD + PRP, and CD + SAI are similar in efficacy. Due to multiple limitations, the results of this study should be interpreted with caution. More high-quality RCTs are needed to further prove our findings.

### Supplementary Information

.

**Additional file 1: Figure S1.** Forest map with results of HHS heterogeneity testing and node splitting method for inconsistency testing. **Figure S2.** Forest map with results of FR heterogeneity testing and node splitting method for inconsistency testing.

## Data Availability

All data generated or analyzed during this study are included in this published article (and its Additional file 1).
