# Supplementary material for: Network meta-analysis of invasive treatment for early-stage osteonecrosis of the femoral head
Source: J Orthop Surg Res. 2024 Jan 3;19:30. doi: 10.1186/s13018-023-04513-x (PMC10765848; doi:10.1186/s13018-023-04513-x)
Supplement: Supplementary file 1 — Additional file 1: Figure S1. Forest map with results of HHS heterogeneity testing and node splitting method for inconsistency testing. Figure S2. Forest map with results of FR heterogeneity testing and node splitting method for inconsistency testing. [file 13018_2023_4513_MOESM1_ESM.docx]

Figure S1 Forest map with results of HHS heterogeneity testing and node splitting method for inconsistency testing
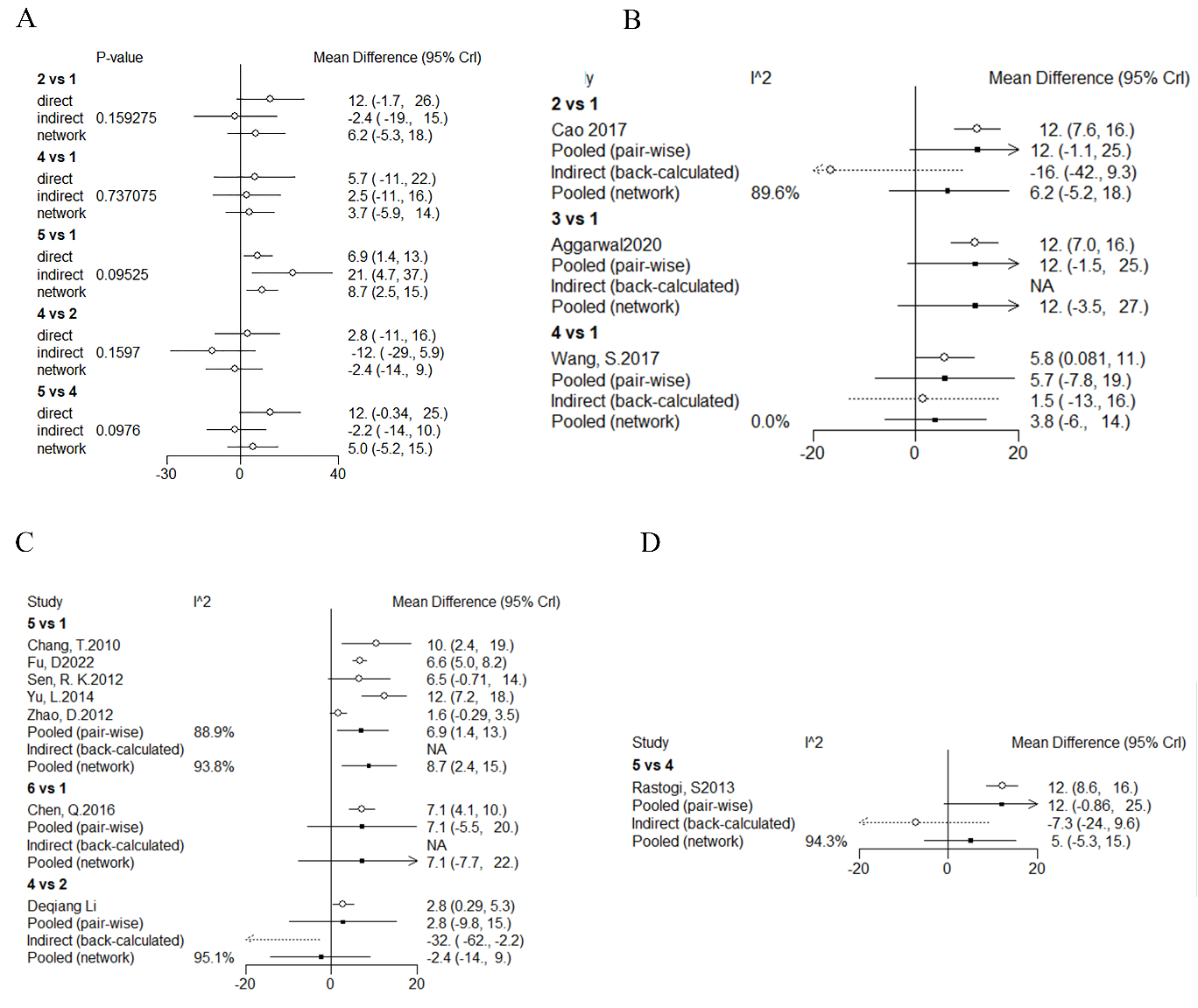


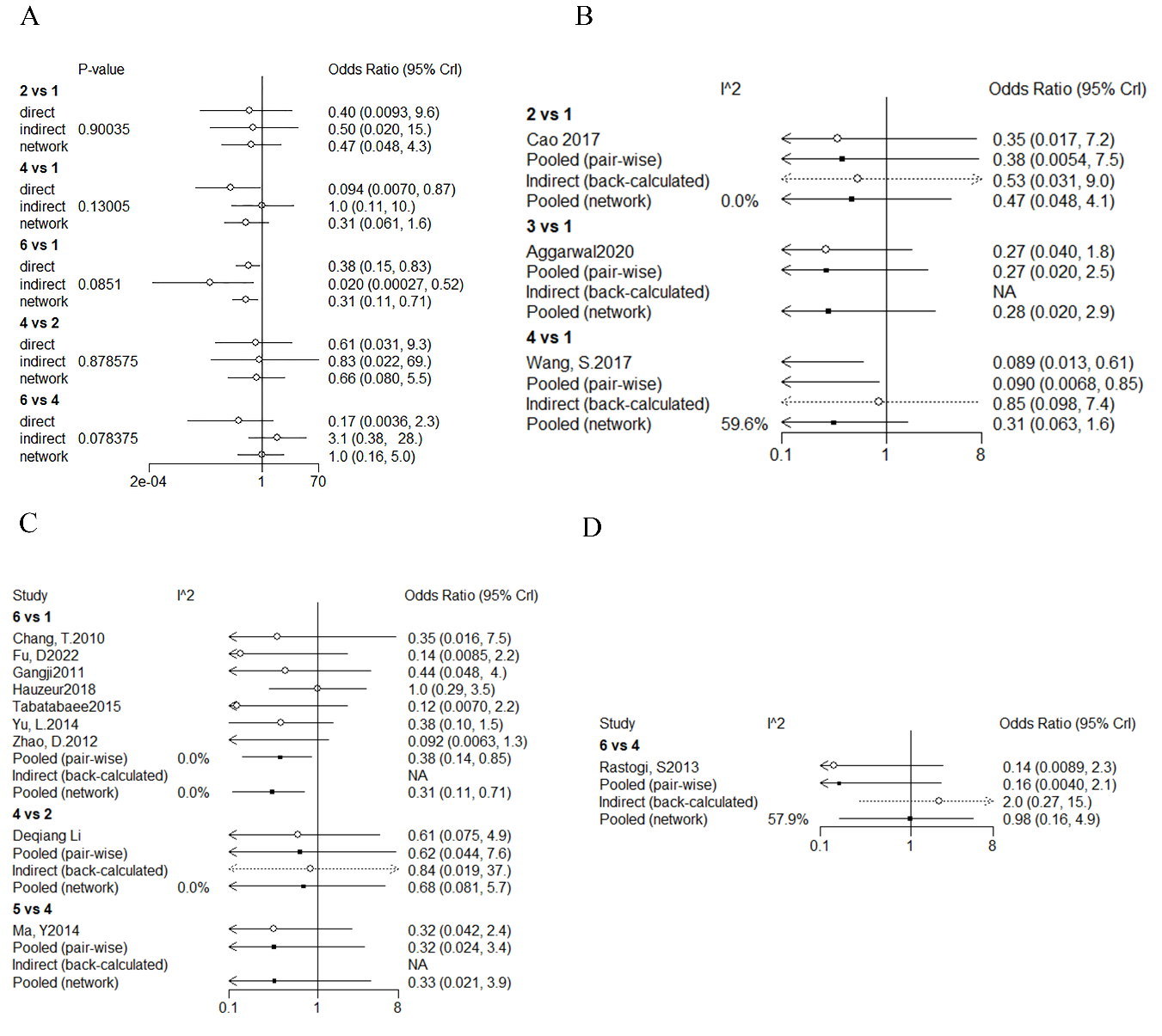


Figure S2 Forest map with results of FR heterogeneity testing and node splitting method for inconsistency testing
